# An Application of the Multivariate Linear Mixed Model to the Analysis of Shoulder Complexity in Breast Cancer Patients

**DOI:** 10.3390/ijerph13030274

**Published:** 2016-03-02

**Authors:** Gholamreza Oskrochi, Emmanuel Lesaffre, Youssof Oskrochi, Delva Shamley

**Affiliations:** 1 Department of Mechanical Engineering an Mathematical Sciences, Oxford Brookes University, Wheatley Campus, Wheatley, Oxford OX33 1HX, UK; 2 Leuven Biostatistics and Statistical Bioinformatics Centre (L-BioStat), Kapucijnenvoer 35 blok D, B-3000 Leuven, Belgium; emmanuel.lesaffre@med.kuleuven.be; 3 Department of Primary Care and Public Health, Imperial College London, Charing Cross Hospital, London W6 8RP, UK; youssof.oskrochi@nhs.net; 4 Clinical Research Centre, University of Cape Town, Old Main Building, L51. Groote Schuur Hospital Observatory, Cape Town 7700, South Africa; delva.shamley@uct.ac.za

**Keywords:** multivariate linear mixed model, correlated random effects, autoregressive of order one

## Abstract

In this study, four major muscles acting on the scapula were investigated in patients who had been treated in the last six years for unilateral carcinoma of the breast. Muscle activity was assessed by electromyography during abduction and adduction of the affected and unaffected arms. The main principal aim of the study was to compare shoulder muscle activity in the affected and unaffected shoulder during elevation of the arm. A multivariate linear mixed model was introduced and applied to address the principal aims. The result of fitting this model to the data shows a huge improvement as compared to the alternatives.

## 1. Introduction

Wide local excision (WLE) with adjuvant radiotherapy is the standard treatment of breast cancer. However, despite the use of less extensive surgery, there is still morbidity affecting the shoulder [1,2,3]. Local radiotherapy has several known effects on lung parenchyma, vascular and connective tissues [4,5,6]. Findings with respect to the latter suggest that thickening of the tissues may restrict movement of surrounding areolar tissues held within fascial planes. This limited ability to expand together with ischaemia due to changes in the vascular network could have an effect on the efficacy of muscle contraction [7,8,9].

Thus far, the exact nature of shoulder morbidity and its relationship to pain has only been described in terms of glenohumeral movement; ignoring the critical effect of scapulo-thoracic motions. Scapular movements are a product of the delicate inter-play between the rotator cuff muscles; thus, deficiency in one can alter all movements due to associations. The pattern of these associations is not known and has been investigated here.

Scapular movements such as abduction, adduction, anterior/posterior tilt, and medial/lateral rotations are a product of the delicate inter-play between the rotator cuff muscles. Deficiency in one may change all other movements. The pattern of these associations is not known and needs to be investigated. 

Four muscles (pectoralis major, serratus anterior, upper trapezius and rhomboid major) acting on the scapula were investigated in patients who had been treated in the last six years for unilateral carcinoma of the breast. Muscle activity was assessed by electromyography (EMG) during abduction and adduction of the ipsilateral (affected) and contralateral (unaffected) sides.

The principal aims of the study were:

(1) to compare shoulder muscle activity in the affected and unaffected shoulder during elevation of the arm;
(2) to explore the relationship between any observed differences in muscle activity and patients report of pain and dysfunction;
(3) to explore the relationship between any observed differences in muscle activity and key clinical variables.

Section 2 introduces the collected data set and its procedure. In Section 3, the multivariate linear mixed models are described which address the above principal aims. Section 4 presents the results of the fit of the model to the data. In Section 5, some final conclusions and a discussion of future analyses are given. 

## 2. Data Set

Two hundred and two patients who had been treated from 2005 to 2010 for unilateral carcinoma of the breast were included in a shoulder morbidity study. The measurement procedure is detailed in our previous work [10]. Patients with any previous history of shoulder or neck problems in either arm were excluded from the study. Humeral elevation in degrees was measured using the Polhemus Fastrak™ motion analysis system, and concurrent EMG readings were taken at 10° increments of humeral elevation of each shoulder for each patient. The average of three EMG readings at each elevation point was taken as a response (dependent) variable for each patient.

All patients filled in a Shoulder Pain and Disability Index (SPADI) questionnaire immediately prior to data collection. The SPADI questionnaire is a known and valid measure of pain and disability for shoulder dysfunction with high levels of sensitivity and reliability [11], it is scored on a visual analogue scale with 13 items (five for pain and eight for disability). All subjects gave their informed consent for inclusion before they participated in the study. The study was conducted in accordance with the Declaration of Helsinki, and the protocol was approved by Oxford Brookes University ethics committee (HREC no: A02,064) 

**Figure 1:**
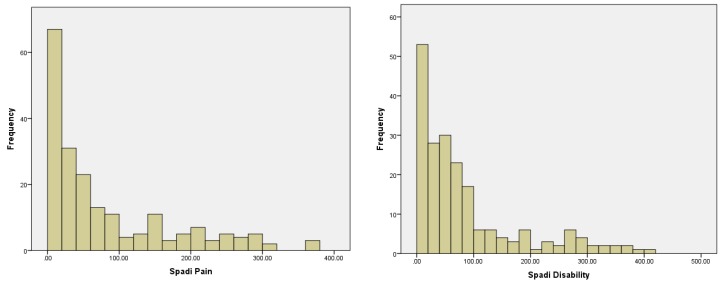
Frequency distribution for pain and disability (*N* = 202).

Pain scores range from a minimum of 0 to a maximum of 500 mm and for disability 0 to 800 mm, where 0 represents no symptoms of pain or disability. Figure 1 represents the distribution of pain and disability scores. Given the skewness of these distributions, each variable is log-transformed in subsequent analyses. 

Key clinical variables include: affected side, dominant hand, degree of arm elevation, treatment protocol (Wide Local Excision (WLE) or other), duration after surgery in days, age in years, physiotherapy and exercise level on affected side, receiving chemotherapy, and the Shoulder Pain and Disability Index [10].

**Figure 2:**
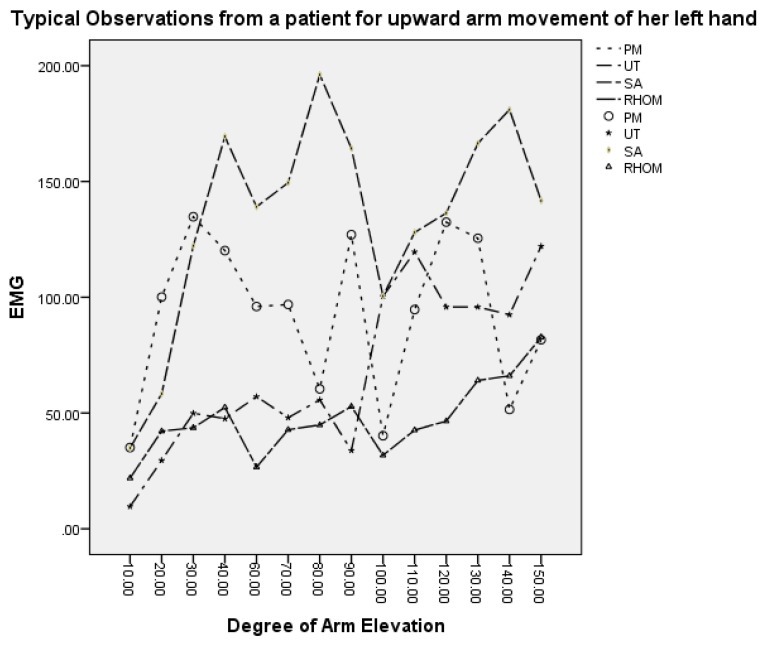
Typical observations from a patient’s arm movement.

Bilateral EMG measurements of the four shoulder muscles were taken for each patient. Each muscle activity is recorded in millivolts (mV) at 10° increments of humeral elevation during upward and downward arm movement. At each increment point, for each shoulder and each movement, four EMG measures were obtained (one for each of the four muscles), creating potentially a correlated or multivariate response structure. Incremental measurements at different arm elevations per muscle created a multivariate response structure that can also be regarded as “repeated measures” dimension of the data. Summarised, four correlated, repeatedly measured responses for each shoulder and each movement were generated. In Figure 2, typical observations of a patient are given, for humeral elevation between 10° and 150°, this figure represents the observations for an upward arm movement of the left arm. This graph clearly shows that the EMG reading at each given point very much depends on the previous reading within the same movement. Hence, this fact should be implemented in the modelling strategy in Section 3.

## 3. Statistical Hypotheses Testing and Statistical Modelling

### 3.1. Statistical Test of Clinical Hypotheses

Shamley *et al.* previously demonstrated [10] that the activities of muscles controlling the movement of the scapula are linked; if one muscle is compromised, then other muscles might become more active to compensate for the lost movement. However, muscle activity can be influenced by several demographic (age, dominance, gender) and clinical (pain, radiotherapy, chemotherapy, *etc.*) variables. In this study, we are interested to explore the previous findings in detail and describe the rationale of whether the clinical variables have the same effect on all muscles, or whether the effect of these variables is different for different muscles under different conditions. Hence, an advanced joint multivariate approach must be implemented to take these associations into consideration whilst investigating the following four main null hypotheses.

*H*_01_: The altered muscle activity is not associated with the patient’s report of pain and dysfunction;
*H*_02_: Clinical risk factors have no effect on the activities of the muscles;
*H*_03_: The effect of clinical risk factors is the same on the affected arm as with that on the unaffected arm.
*H*_04_: The activity of the four key muscles acting at the scapula on the affected arm is the same as from those acting on the unaffected arm.

### 3.2. The Multivariate Linear Mixed Model

Let ***Y**_iksm_* denote the *n_i_* × 1 vector of EMG readings at different humeral elevation points from the i^th^ patient (*i* = 1,…, *N*), *n_i_* is the number of elevation points for that patient on the *k*^th^ muscle (*k* = 1,…, 4), of the affected arm (*s = 1*) or unaffected arm (*s = 2*) during upward (*m = 1*) or downward (*m = 2*) movements. 

We assume for the *n_i_* measurements, ***Y**_iksm_*, a multivariate normal distribution over the elevations. It is in fact the usual multivariate multiple regression, *i.e.*,:

(1) $$Y_{iksm}∼N\left(X_{iksm}\beta_{ksm},\Sigma_{k}\right)$$

where ***X**_iksm_* is a *n_i_* × *p* matrix of covariates (*p* is the number of covariates), ***β**_ksm_* is a *p* × 1 vector of regression coefficients and Σ_*k*_ is a *n_i_* × *n_i_* covariance matrix. 

Obviously, when the humeral elevations are closer, the correlation should be stronger, therefore, after testing a few other correlation structures, we sensibly assumed an autoregressive structure of order one, *i.e.*, AR(1) for Σ_*k*_, *i.e.*,

(2) $$\Sigma_{k}=\sigma_{k}^{2}\left(\begin{array}{l} \begin{array}{cccc} 1 & \rho_{k} & \rho_{k}^{2} & ‥\\ \rho_{k} & 1 & \rho_{k} & \rho_{k}^{2}\\ \rho_{k}^{2} & \rho_{k} & 1 & \rho_{k}\\ : & : & : & : \end{array}\begin{array}{c} \rho_{k}^{n_{i}-1}\\ ‥\\ ‥\\ : \end{array}\\ \begin{array}{cccc} \rho_{k}^{n_{i}-1} & ‥ & \ldots \rho_{k} & 1 \end{array} \end{array}\right)$$

A simplifying assumption could be to assume that the responses are independent among patients and muscles, that is

(3) $$Y_{ik}=\left[\begin{array}{c} Y_{ik11}\\ Y_{ik12}\\ Y_{ik21}\\ Y_{ik22} \end{array}\right]∼N\left[\left(\begin{array}{c} X_{ik11}\beta_{k11}\\ X_{ik12}\beta_{k12}\\ X_{ik21}\beta_{k21}\\ X_{ik22}\beta_{k22} \end{array}\right),\Sigma_{kk}=\left(\begin{array}{cccc} \Sigma_{k} & 0 & 0 & 0\\ 0 & \Sigma_{k} & 0 & 0\\ 0 & 0 & \Sigma_{k} & 0\\ 0 & 0 & 0 & \Sigma_{k} \end{array}\right)\right]$$

It is generally unrealistic to assume that all important risk factors (covariates) are measured and included in the model explanatory matrix (***X**_iksm_*). The unobserved or unmeasured risk factors of a muscle, which are usually known as the “unobserved muscle’s specific effect”, causes the measurements of a muscle to be more correlated. To improve Model (3), one could introduce a “muscle specific effect” or random effect into the model. This term usually is known as frailty, so that, conditional on this muscle’s specific effect, ***ν**_ik_*, we can assume

(4) $$Y_{ik}|v_{k}\sim N\left(X_{ik}\beta_{k}+v_{ik},\Sigma_{kk}\right)$$

with, ***X**_ik_*, the stacked design matrix $X_{ik}^{T}=\left[X_{ik11}^{T}X_{ik12}^{T}X_{ik21}^{T}X_{ik22}^{T}\right]$ and $\beta_{k}^{T}=\left[\beta_{k11}^{T},\beta_{k12}^{T},\beta_{k21}^{T},\beta_{k22}^{T}\right]$. 

Coull and Agresti [12] presented similar models for multivariate binomial logit-normal. Fieuws *et al.* [13] discussed similar models in more detail.

Furthermore, we assumed that the frailty terms of different muscles are correlated. That is, we assumed that the vector of frailty terms $\nu_{i}^{T}=\left(\nu_{i1},\nu_{i2},\nu_{i3},\nu_{i4}\right)$ has the following distribution:

(5) $$\nu_{i}=\left[\begin{array}{c} \nu_{i1}\\ \nu_{i2}\\ \nu_{i3}\\ \nu_{i4} \end{array}\right]∼N\left[\left(\begin{array}{c} 0\\ 0\\ 0\\ 0 \end{array}\right),D\;=\;\left(\begin{array}{cccc} d_{11} & d_{12} & d_{13} & d_{14}\\ d_{12} & d_{22} & d_{23} & d_{24}\\ d_{13} & d_{23} & d_{33} & d_{34}\\ d_{14} & d_{24} & d_{34} & d_{44} \end{array}\right)\right].$$

We assume that the joint model of $Y_{i}^{T}=\left(Y_{i1},Y_{i2},Y_{i3},Y_{i4}\right)$ follows a conditional multivariate normal distribution

(6) $$Y_{i}|\nu_{i}∼N\left[\left(\begin{array}{c} X_{i1}\beta_{1}+\nu_{i1},\\ X_{i2}\beta_{2}+\nu_{i2},\\ X_{i3}\beta_{3}+\nu_{i3},\\ X_{i4}\beta_{4}+\nu_{i4}, \end{array}\right),\Sigma =\left(\begin{array}{cccc} \Sigma_{1} & 0 & 0 & 0\\ 0 & \Sigma_{2} & 0 & 0\\ 0 & 0 & \Sigma_{3} & 0\\ 0 & 0 & 0 & \Sigma_{4} \end{array}\right)\right]$$

with the normal distribution of the frailty vector which adds the covariance structure to the frailties across muscles. The joint model defined by Model (6) is a multivariate linear mixed model with multivariate random effects *ν_i_*.

As for the univariate linear mixed model with a univariate normal random effect, the marginal likelihood in Model (6) is also analytically tractable. We used SAS™ [14] 9.2, PROC MIXED (SAS, Cary, NC, USA) to estimate the model parameters.

In this model, the correlation structure among the muscles is presented by the correlation structure among their specific frailties given by the variance-covariance matrix (*D*); if the muscles are not significantly correlated, all off-diagonal entities in the variance-covariance matrix *D* will be zero. Significant non-zero off-diagonal entities of variance-covariance matrix *D* are showing the correlations among the muscles. 

## 4. Empirical Results

### 4.1. Model Comparison

Table 1 presents the result of analyzing the natural log transformation of EMG activities of each muscle using Model (3), *i.e.*, a multivariate normal distribution for ln(*emg_iksm_*) = *Y_iksm_* with block diagonal variance-covariance matrix of *Σ_k_*. The likelihood ratio test suggests that the effect of arm movement (MOVE_UP/MOVE_DOWN) is best presented by a dummy variable indicating different intercepts for the upward and downward movement. Interactions between arm movements with all other clinical risk factors (covariates) were not collectively significant at the 5% level.

Contrariwise, the effect of affected shoulder and unaffected shoulder cannot be modelled by considering only different intercepts for affected/unaffected shoulder. This is due to the fact that some of the risk factors are specific to the affected shoulder, such as physiotherapy and exercise. This implies that the effect of clinical risk factors might well be different on the affected shoulder compared to the unaffected shoulder. Therefore, a full interaction model (all main effects and all interactions) initially was employed to assess the effects of the affected shoulder on all clinical risk factors; the highly insignificant interactions were subsequently dropped from the model. The likelihood ratio test suggests that the included interactions are highly significant (change in deviance of 717.2 for 32 degrees of freedom). 

The values in bold correspond to significant effects (at the 5% level) for each muscle. The last column (“*p*-value”) indicates whether the clinical risk factor is collectively (the effect on all four muscles collectively with four degrees of freedom) significant or not. 

A significant value for the interaction terms shows that, over and above the overall effect of the clinical risk factor on electrical activity of the muscles, the effect of clinical risk factor on the affected side is different to the unaffected side. The autoregressive correlation (*ρ_k_*) and the variance of each muscle ($\sigma_{k}^{2}$) are also presented.

The result of fitting a multivariate normally distributed model with AR(1) structure for *Σ_k_*, presented in Table 1, controls the autoregressive structure of the measurements. The high positive value of ***ρ*** shows that the electrical activities are more alike at humeral elevation angles that are closer together. 

This model not only ignores associations among the four muscles but also ignores any possible associations between measurements of the same muscle, *i.e.*, affected/unaffected shoulders and downwards/upwards movement. In other words, this model assumes independence between the four muscles and between all observations of the same muscle at the same humeral elevation. This is not a realistic assumption as the four muscles are from the same individual and are likely to be correlated, and all measurements of the same muscle at the same humeral elevation are also likely to be correlated as they are from the same muscle. Table 2 is the result of fitting a univariate linear mixed Model (4) with independent random intercept *ν_k_* across muscles and residual block diagonal variance-covariance matrix with blocks *Σ_k_* assumed to follow the auto-regressive process of order one. Model (4) controls the association between the measurements of the same muscles at the same humeral elevation point by including a muscle specific random effect into the model. 

This model suggests that humeral elevation of the arm, the upwards move, affected side and left shoulder will increase the electrical activity generally for all four muscles irrespective of which side is affected, whilst treatment with wide local excision and receiving chemotherapy are associated with generally decreased electrical activity. 

Interaction analysis suggests that the electrical activity is significantly different for duration after surgery and SPADI pain. Doing exercise and having physiotherapy on the affected shoulder are significantly associated with electrical activity of the affected muscles. The deviance (−2 × log likelihood) for this model is 27,645.1. The change in deviance compared to Model (3) is 1536.1 for four degrees of freedom (*p*-value < 0.0001), which is highly statistically significant. 

Univariate Linear Mixed Model with AR(1) structure for *Σ_k_* controls the autoregressive structure of the measurements and accounts for possible associations between measurements of the same muscle at the same elevation point. The disadvantage of Model (4) is that it ignores any possible associations among the four muscles within patients. Table 3 is the result of fitting a full multivariate linear mixed model with AR(1) structure for *Σ_k_* given in Model (6).

The result of fitting a full multivariate Linear Mixed Model with AR(1) structure for *Σ_k_*, as expected, is almost similar to that of Model (4). This model suggests that humeral elevation of the arm, the upwards move, affected side, left shoulder and longer duration from the surgery will increase the electrical activity generally for all four muscles irrespective of the side affected—whilst treatment with wide local excision and receiving chemotherapy are associated with generally decreased electrical activity 

Interaction analysis suggests that electrical activity is significantly different for duration after surgery and SPADI pain. Once again, doing exercise and having physiotherapy on the affected shoulder are significantly associated with electrical activity of the affected muscles. SPADI pain increases the muscle activity in the affected arm, but the affected arm slightly dilutes the positive effect of duration since surgery on the muscle activity. The deviance for this model is 26833.2. The change in deviance compared to Model (4) is 811.9 for six degrees of freedom, and, compared to Model (3), the change in deviance is 2348 for 10 degrees of freedom. Both suggest statistically significant improvement (both *p*-values < 0.0001) in model fitting. 

Hence, using a multivariate normal distribution for natural log of muscle activities with AR(1) structure for *Σ_k_*, for each muscle and ignoring the existing associations between the measurements leads to unrealistic inferences for important clinical variables.

Applying a univariate Linear Mixed Model with AR(1) structure for *Σ_k_* (4) while ignoring the associations between muscle specific effects leads to clinically much more sensible parameter estimates. Subsequently, comparing the likelihoods of Models (3) and (4) confirms the presence of strong muscle specific effects.

A full multivariate Linear Mixed Model with AR(1) structure for *Σ_k_* (6) assesses the presence of significant association between muscle-specific random effects. A deviance difference of 811.9 for six degrees of freedom suggests the use of a joint multivariate linear mixed model with residual block diagonal variance-covariance matrix, which assumed to following auto-regressive process of order one, is more appropriate. 

In this model, the associations between measurements of the same movement at different elevation points is given by ***ρ*** for each muscle. The estimated value of ***ρ*** is about 0.8, which shows a strong association between measurements of the same movement. However, the associations between muscles where modelled via their unobserved specific random effects. The estimated variance-covariance matrix of this association is given in the following matrix $\hat{D}$:

(7) $$\hat{D}\;=\;\left(\begin{array}{cccc} 0.33 & & & \\ 0.23 & 0.38 & & \\ 0.21 & 0.18 & 0.29 & \\ 0.24 & 0.25 & 0.22 & 0.35 \end{array}\right)$$

A likelihood ratio test comparing Models (4) and (6) shows that the associations between muscles are very important and cannot be ignored. The estimated variance-covariance matrix $\hat{D}$ indicates that all estimated off-diagonal entities are positive, which demonstrates that all correlations among the muscles are significantly positive.

### 4.2. Statistical Inference of Clinical Hypotheses

The result of Model (6), which is presented in Table 3, can be explored and refitted to assess the main clinical hypotheses.

*H*_01_: The activity of four key muscles acting at the scapula on the affected arm is not different to those acting on the unaffected arm.

The null hypothesis *H*_01_ should be rejected as the activity of four key muscles acting at the scapula on the affected arm is significantly different to the unaffected arm. The gain in deviance between models with and without affected side interactions is 689.6 for 16 degrees of freedom returning a *p*-value < 0.0001, highly statistically significant.

*H*_02_: Altered muscle activity is not associated with patients’ report of pain or dysfunction.

SPADI pain has a significant effect on activity of the affected arm *p*-value < 0.0001); however, report of pain and dysfunction had no significant effect in general.

*H*_03_: Clinical risk factors have no significant effect on the activity of muscles.

Clinical risk factors have a significant effect on muscle activities. For instance, WLE treatment (*p*-value = 0.027) and chemotherapy (*p*-value = 0.021), compared to their alternatives, have adverse effects on all muscle activities. 

*H*_04_: The effects of clinical risk factors are not different on the affected arm as compared to the unaffected arm. The effects of clinical risk factors on muscle activity are different on the affected arm compared to the unaffected arm. For example, reports of pain and dysfunction (SPADI pain) had no significant effect on the unaffected arm (*p*-value = 0.422), but increasing SPADI pain is associated with increased muscle activity in the affected arm (*p*-value < 0.0001). Duration since surgery is associated with decreased muscle activity in the affected arm as compared to the unaffected arm (*p*-value < 0.0001). 

### 4.3. Checking Model Assumptions

**Figure 3:**
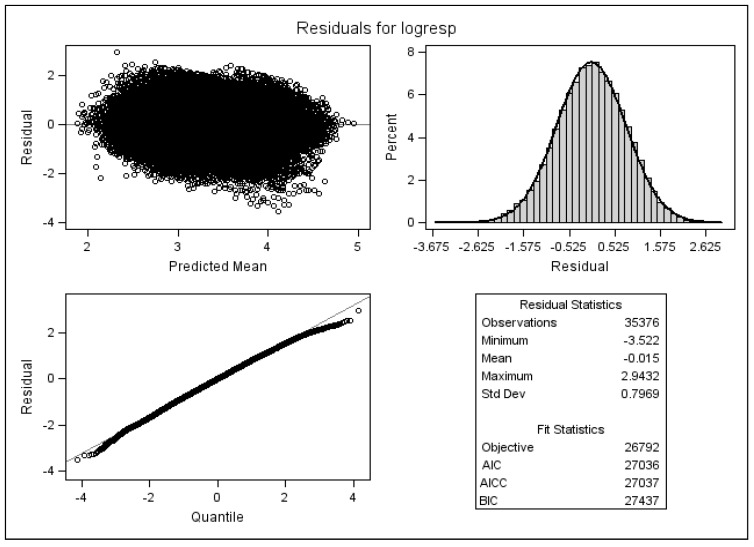
Standardised residuals of the multivariate mixed linear model.

Figure 3 is the result of a diagnostic analysis, and it shows the standardised residuals of the full multivariate mixed linear model with AR(1) variance-covariance structure. It is clear that most of the standardised residuals have an absolute value of less than 2.0, which could have easily occurred by chance. It is expected that 5% of standardised residuals should be greater than 2.0 in absolute value according to expected standard normal distribution of the standardised residuals. Hence, Figure 3 does not show any serious diversion from normality for residuals of the implemented multivariate linear mixed model given by Model (6). 

**Figure 4:**
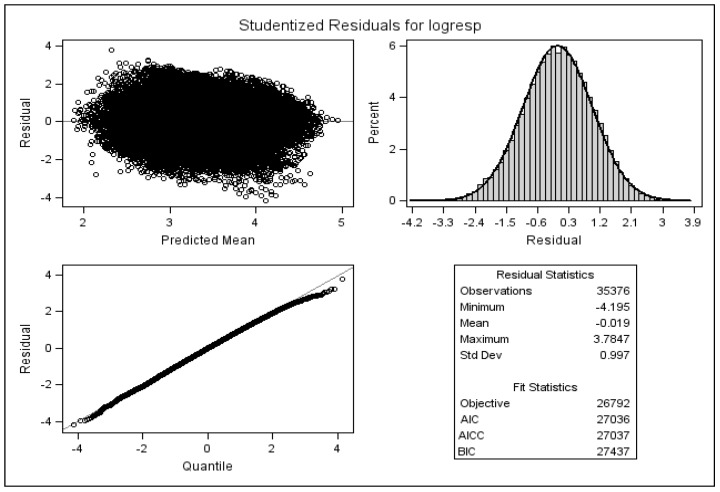
Standardised residuals with the *i^t^*^h^ observation removed.

Figure 4 may potentially detect influential observations. Here, residuals are presented similar to the standardised residuals except that they are calculated after deleting the *i*^th^ observation. In other words, the figure shows the difference between the observed response values and the predicted response values excluding the *i*^th^ observation from the regression.

The comparison between the two figures does not suggest any major influential observation that if removed has significant effect on parameter estimates. 

## 5. Discussions

Our results demonstrated that the affected side is significantly different in terms of muscle activity than the unaffected side. This is understandable given the nature of the disease and treatment. The results of our model also demonstrate, however, several other important points.

Firstly, with respect to personal factors, only pain appears to play a significant role. Whilst no effect was observed with respect to SPADI pain in the unaffected arm, it was associated with increased muscle activity in the affected arm. This phenomenon has been previously reported and is hypothesised to be a functional adaptation in order to limit movements of the painful muscle, hence reduce overall pain [15].

Hand dominance had no significant effect on muscle activity, regardless of the operated side correlating well with other studies demonstrating either no difference in female subjects [16] or only significance in a few movements, primarily the flexion [17,18,19]. Still independent of the operative side, there is also greater activation and activity in shoulder muscles during abduction of the arm compared to adduction, with the magnitude of activity being proportional to the extent of abduction. The significant difference between abduction and adduction as displayed by the model is naturally expected due to the nature of gravity assisting adduction and thus activating of fewer muscle spindles. We hypothesise that if adduction was performed against resistance such as to simulate the force of gravity which muscles experience during abduction, then activity would be similar. The significance of these findings between healthy volunteers and breast cancer survivors should be assessed in a case-control study.

With respect to clinical factors, previous work had already demonstrated that surgical breast cancer treatment resulted in shoulder morbidity [10], with recent work also showing mastectomy causing greater degrees than WLE [20]. This study of WLE, controlling for chemotherapy and radiotherapy effects, also demonstrated a significant adverse effect on all muscles independent of the affected side, similar to previously reported results. 

The effects of various variables on muscles are summarized in Table 4. The effect of time since surgery on muscle activity was not significant per muscle, although, overall, its effect was significant. The effect of chemotherapy similarly shows significant reduction in overall muscle activity on the ipsilateral side, although, individually, the difference was only significant for the serratus anterior. Chemotherapy induced peripheral neuropathy (CIPN) is a well-known complication of adjuvant therapy, and its incidence is closely linked to the agents used. Although primarily a sensory neuropathy, motor complications have also been reported [21] with greater impact on quality of life [22]. Taxanes, prolific agents used in breast cancer treatment [23], have been implicated in reduced compound muscle action potentials and myopathy, although less frequently than their sensory effects [24]. With respect to the observed difference on the contralateral side with serratus anterior, we could hypothesise that, due to the length of the long thoracic nerve, it may be more at risk from the neurotoxic effects of the chemotherapy agents. This is not without precedence, as the sural nerve, another similarly long nerve, has been reported as being particularly at risk of CIPN with platinum and paclitaxel based compounds [25].

With respect to rehabilitation, our model indicates that pain, physiotherapy and exercise result in an overall significant increase in muscle activity on the affected shoulder (Table 5). Specifically, exercise results in independent increases for the pectoralis major, upper trapezius and serratus anterior whilst physiotherapy results in similar findings for the latter two aforementioned muscles. With respect to the affected side, only our findings are summarised in Table 5. 

Physiotherapy, either currently or at any point, was shown to improve muscle activity on the affected shoulder significantly. Multiple previous studies have demonstrated the beneficial effect of physiotherapy on shoulder function; maintenance of movement ranges, reduced pain, improved muscle strength and overall better quality of life are all reported [1,26,27].

## 6. Conclusions

Shoulder EMG activity and, therefore, muscle activation depends on both clinical and personal variables. Clinically the use of chemotherapy, WLE and time since surgery all decrease muscle activation, whilst physiotherapy increases it. Pain, a personal subjective influence, serves to increase muscle activation and is likely to limit movement and prevent further pain. This work builds on previously published data and offers new insights into the variables that affect shoulder morbidity with an effective model. This study further adds to the understanding of modelling multi-dimensional data and illustrates the risk of ignoring potential associations in the data structure. Several modelling approaches were discussed and compared, and the more suitable analysis method was identified and applied to the data. 

## Figures and Tables

**Table 1:** Results of the analysis using a multivariate normal distribution with autoregressive of order one (AR(1)) structure for *Σ_k_*.

Clinical Measures	Parameter Estimates				*p*-Value
	Ln(PM)	Ln(UT)	Ln(SA)	Ln(RH)	Overall Effect
Intercept	2.9812	3.07	2.7905	2.1944	<0.0001
Humeral elevation × 100	0.5053	0.5868	0.8403	0.7097	<0.0001
Move up	0.1175	0.287	0.1286	0.1746	<.0001
Affected	−0.1897	0.02896	0.4124	−0.0995	0.1855
Handleft	0.2908	0.1677	0.1959	0.1686	<0.0001
Dominant	0.07338	0.1233	0.07865	0.07744	0.4888
Agex100	−0.00037	0.4736	−0.503	0.4888	0.0386
Duration × 100	−0.01	0.0024	0.0119	0.0125	0.0286
Spadipai × 100	−0.146	−0.156	−0.061	−0.095	0.0005
Spadidis × 100	0.0591	0.0726	0.0707	−0.042	0.1284
Wle	−0.2556	−0.1913	−0.2544	−0.2267	<0.0001
Chemocat	−0.1107	−0.0803	−0.4658	−0.1233	<0.0001
Inter_humeral elevation * × 100	0.0161	0.0837	0.019	0.1194	0.2037
Inter_dominant *	−0.2211	−0.1212	−0.3452	−0.2614	<0.0001
Inter_duration * × 100	0.0037	−0.004	−0.021	−0.007	0.1410
Inter_spadipai * × 100	0.1083	0.1046	−0.051	0.0948	0.0239
Exercise 6 m **	−0.00577	−0.0644	−0.05941	0.01678	0.0349
Exercise now **	0.06808	0.05504	0.08655	0.01458	<0.0001
Physio now **	0.307	0.5523	0.1583	0.3082	0.1207
Physio ever **	−0.01245	−0.0663	0.1235	−0.1439	0.4765
***ρ***	0.89	0.92	0.90	0.91	N/A
**σ**^2^	0.62	0.73	0.61	0.63	N/A
–2 log likelihood of the model with interaction terms –2 log likelihood of the model without interaction					29,181.2 29,898.4

* Interaction with affected side, ** Only on affected side.

**Table 2:** Result of the analysis using univariate Linear Mixed Model with AR(1) structure for *Σ_k_*.

Clinical Measures	Parameter Estimates				*p*-Value
	Ln(PM)	Ln(UT)	Ln(SA)	Ln(RH)	Overall Effect
Intercept	3.0631	3.1468	2.8339	2.2456	<0.0001
Humeral elevation × 100	0.4999	0.5733	0.8288	0.6937	<0.0001
Move up	0.1357	0.2985	0.136	0.2008	<0.0001
Affected	–0.06145	0.07579	0.5562	0.1457	0.0113
Handleft	0.2825	0.154	0.199	0.1627	<0.0001
Dominant	0.07379	0.1044	0.07393	0.07766	0.6258
Agex100	–0.088	0.4317	−0.542	0.4361	0.5757
Duration × 100	–0.011	0.0022	0.0113	0.0119	0.2323
Spadipai × 100	–0.147	–0.155	–0.063	–0.104	0.1053
Spadidis × 100	0.0532	0.0676	0.0589	–0.035	0.8000
Wle	–0.2831	−0.199	−0.2626	–0.2262	<0.0001
Chemocat	–0.1611	–0.1034	–0.4759	−0.1365	0.0231
Inter_humeral elevation * × 100	0.0029	0.0774	0.0047	0.1091	0.1146
Inter_dominant *	–0.2326	–0.1192	–0.3466	–0.2746	0.0654
Inter_duration * × 100	0.0022	–0.007	–0.024	−0.011	<0.0001
Inter_spadipai * × 100	0.1042	0.1195	−0.023	0.1221	<0.0001
Exercise 6 m **	–0.02449	–0.04658	–0.05968	0.003809	0.0142
Exercise now **	0.06914	0.01997	0.05287	–0.03337	<0.0001
Physio now **	0.01201	0.4788	0.1107	0.5951	0.0060
Physio ever **	0.002231	–0.1651	0.01827	–0.2481	0.0163
***ρ***	0.79	0.85	0.82	0.80	N/A
**σ**^2^	0.31	0.37	0.33	0.29	N/A
Random effect variance	0.33	0.38	0.28	0.35	N/A
–2 log likelihood of the model with interaction terms –2 log likelihood of the model without interaction					27,645.1 27,862.8

* Interaction with affected side, ** Only on affected side.

**Table 3:** Result of the analysis using multivariate Linear Mixed Model with AR(1) structure for *Σ_k_*.

Clinical Measures	Parameter Estimates				*p*-Value
	Ln(PM)	Ln(UT)	Ln(SA)	Ln(RH)	Overall Effect
Intercept	3.0662	3.1566	2.8413	2.2559	<0.0001
Humeral elevation × 100	0.5012	0.5761	0.8314	0.694	<0.0001
Move_up	0.1357	0.299	0.1362	0.201	<0.0001
Affected	–0.03653	0.1271	0.5837	0.1368	0.0054
Handleft	0.2837	0.1537	0.1983	0.1608	<0.0001
Dominant	0.07391	0.1057	0.07586	0.07687	0.8796
Age × 100	–0.093	0.4211	–0.549	0.4292	0.1775
Duration × 100	–0.011	0.0021	0.0112	0.0119	0.0227
Spadipai × 100	–0.148	–0.155	–0.064	–0.103	0.4220
Spadidis × 100	0.0533	0.0679	0.0604	–0.037	0.5270
Wle	–0.283	–0.2028	–0.2668	–0.2306	0.0272
Chemocat	–0.1598	–0.1078	–0.4812	–0.1446	0.0205
Inter_humeral elevation * × 100	5.07E-04	0.0737	0.0036	0.1096	0.1219
Inter_dominant *	–0.2347	–0.1247	–0.3505	–0.2764	0.2958
Inter_duration * × 100	0.0015	−0.008	–0.024	–0.011	<0.0001
Inter_spadipai * × 100	0.1115	0.1211	–0.023	0.1212	<0.0001
Exercise 6 m **	–0.02175	–0.05333	–0.06286	0.006382	0.0049
Exercise now **	0.05879	0.01727	0.05089	–0.03237	<0.0001
Physio now **	0.08553	0.4907	0.1158	0.5001	0.0177
Physio ever **	–0.0337	–0.1669	0.02314	–0.2484	0.0121
***ρ***	0.79	0.85	0.82	0.80	N/A
**σ**^2^	0.31	0.37	0.33	0.29	N/A
Random effect variance	0.33	0.38	0.29	0.35	N/A
–2 log likelihood of the model with interaction terms –2 log likelihood of the model without interaction					26,833.2 27,522.8

* Interaction with affected side, ** Only on affected side.

**Table 4:** Factors that significantly affect muscle activity, irrespective of affected shoulder.

Variable	EMG Effect (Significant)			
	Pectoralis Major	Upper Trapezius	Serratus Anterior	Rhomboid
Humeral Elevation	↑ *	↑ *	↑ *	↑ *
Left *vs.* Right Shoulder	↑ *	↑ *	↑ *	↑ *
Time Since Surgery (DURATION)	**↓**	**↑**	**↑**	**↑**
WLE (*vs.* Other Treatment Modalities)	↓ *	↓ *	↓ *	↓ *
Chemotherapy	**↓**	**↓**	↓ *	**↓**

* indicates, significant at the 5% level.

**Table 5:** Significant factors affecting muscle activity on the affected shoulder as compared to the unaffected shoulder.

Variable	EMG Effect (Significant)			
	Pectoralis Major	Upper Trapezius	Serratus Anterior	Rhomboid
Increasing Pain Score	↑ *	↑ *	**↓**	↑ *
Exercise Last 6 Months	**↓**	↓ *	↓ *	**↑**
Current Exercise	↑ *	**↑**	↑ *	↓ *
Current Physiotherapy	**↑**	↑ *	**↑**	↑ *
Time Since Surgery	**↑**	**↓**	↓ *	↓ *
